# NRF1-mediated microglial activation triggers high-altitude cerebral edema

**DOI:** 10.1093/jmcb/mjac036

**Published:** 2022-06-15

**Authors:** Xueting Wang, Guijuan Chen, Baolan Wan, Zhangji Dong, Yan Xue, Qianqian Luo, Dan Wang, Yapeng Lu, Li Zhu

**Affiliations:** Institute of Special Environmental Medicine, Nantong University, Nantong 226019, China; Co-Innovation Center of Neuroregeneration, Jiangsu Key Laboratory of Neuroregeneration, Nantong University, Nantong 226019, China; Institute of Special Environmental Medicine, Nantong University, Nantong 226019, China; Co-Innovation Center of Neuroregeneration, Jiangsu Key Laboratory of Neuroregeneration, Nantong University, Nantong 226019, China; Institute of Special Environmental Medicine, Nantong University, Nantong 226019, China; Co-Innovation Center of Neuroregeneration, Jiangsu Key Laboratory of Neuroregeneration, Nantong University, Nantong 226019, China; Co-Innovation Center of Neuroregeneration, Jiangsu Key Laboratory of Neuroregeneration, Nantong University, Nantong 226019, China; Key Laboratory of Neuroregeneration of Jiangsu and Ministry of Education, Nantong University, Nantong 226019, China; Institute of Special Environmental Medicine, Nantong University, Nantong 226019, China; Co-Innovation Center of Neuroregeneration, Jiangsu Key Laboratory of Neuroregeneration, Nantong University, Nantong 226019, China; Institute of Special Environmental Medicine, Nantong University, Nantong 226019, China; Co-Innovation Center of Neuroregeneration, Jiangsu Key Laboratory of Neuroregeneration, Nantong University, Nantong 226019, China; Institute of Special Environmental Medicine, Nantong University, Nantong 226019, China; Co-Innovation Center of Neuroregeneration, Jiangsu Key Laboratory of Neuroregeneration, Nantong University, Nantong 226019, China; Institute of Special Environmental Medicine, Nantong University, Nantong 226019, China; Co-Innovation Center of Neuroregeneration, Jiangsu Key Laboratory of Neuroregeneration, Nantong University, Nantong 226019, China; Institute of Special Environmental Medicine, Nantong University, Nantong 226019, China; Co-Innovation Center of Neuroregeneration, Jiangsu Key Laboratory of Neuroregeneration, Nantong University, Nantong 226019, China

**Keywords:** high-altitude cerebral edema, hypoxia, microglia, inflammation, nuclear respiratory factor 1, endocytosis

## Abstract

High-altitude cerebral edema (HACE) is a potentially fatal encephalopathy associated with a time-dependent exposure to the hypobaric hypoxia of altitude. The formation of HACE is affected by both vasogenic and cytotoxic edema. The over-activated microglia potentiate the damage of blood–brain barrier (BBB) and exacerbate cytotoxic edema. In light with the activation of microglia in HACE, we aimed to investigate whether the over-activated microglia were the key turning point of acute mountain sickness to HACE. In *in vivo* experiments, by exposing mice to hypobaric hypoxia (7000 m above sea level) to induce HACE model, we found that microglia were activated and migrated to blood vessels. Microglia depletion by PLX5622 obviously relieved brain edema. In *in vitro* experiments, we found that hypoxia induced cultured microglial activation, leading to the destruction of endothelial tight junction and astrocyte swelling. Up-regulated nuclear respiratory factor 1 (NRF1) accelerated pro-inflammatory factors through transcriptional regulation on nuclear factor kappa B p65 (NF-κB p65) and mitochondrial transcription factor A (TFAM) in activated microglia under hypoxia. NRF1 also up-regulated phagocytosis by transcriptional regulation on caveolin-1 (CAV-1) and adaptor-related protein complex 2 subunit beta (AP2B1). The present study reveals a new mechanism in HACE: hypoxia over-activates microglia through up-regulation of NRF1, which both induces inflammatory response through transcriptionally activating NF-κB p65 and TFAM, and enhances phagocytic function through up-regulation of CAV-1 and AP2B1; hypoxia-activated microglia destroy the integrity of BBB and release pro-inflammatory factors that eventually induce HACE.

## Introduction

High-altitude cerebral edema (HACE) is a rare and potentially fatal encephalopathy caused by a time-dependent exposure to high-altitude thin air. Most experts consider HACE as a clinical and pathological extension of acute mountain sickness (AMS) (Jensen and Vincent, 2020). The incidence of HACE is ∼0.5%–2% (Hackett and Roach, 2004; Wu et al., 2006), which is more frequent at an altitude of 4500–5500 m (Imray et al., 2010). HACE is associated with the rapid ascension to a high-altitude environment, a time-dependent exposure to the hypobaric hypoxia (HH) of altitude, infection, emotion, and genetic factors (Armstrong, 2020). HACE may progress rapidly to coma and death within 24 h as a result of brain herniation, posing a hazard to the lives and health of the garrison and tourists at high altitudes. However, the exact mechanism of the development of HACE is not fully understood, making clinical prevention and treatment of HACE challenging.

Microglia, a type of tissue-resident macrophage in the brain, benefit the homeostatic of the brain (Kettenmann et al., 2011; Li and Barres, 2018). The polarization state of microglia plays dual roles in the integrity of the blood–brain barrier (BBB) (Haruwaka et al., 2019; Kang et al., 2020). Resting microglia promote the integrity of BBB, while activated microglia are recruited to blood vessels due to chemotaxis, phagocytize the tight junction of vascular endothelium, and eventually up-regulate vascular permeability (Liu et al., 2018). Depletion of microglia by PLX5622 effectively slows down the damage of BBB caused by cerebral hemorrhage or hypertension (Kerkhofs et al., 2020; Willis et al., 2020). Furthermore, hypoxia triggers a pro-inflammatory response in microglia, which benefits astrocyte swelling by up-regulating aquaporin 4 (AQP4), the hallmark protein of cytotoxic edema in HACE (Wang et al., 2018; Zhao et al., 2018). Based on the existence of both vascular edema and cytotoxic edema in HACE (Hackett, 1999; Kallenberg et al., 2007; Zhou et al., 2017; Urushida et al., 2021), we speculate that over-activation of microglia triggers AMS progressing to HACE.

Through RNA sequencing (RNA-seq), an important transcription factor, nuclear respiratory factor 1 (NRF1) was screened out, which was highly expressed in hypoxia-activated microglia. NRF1 is known to regulate the expression of mitochondrial respiratory complex (Bergeron et al., 2001; Piantadosi and Suliman, 2006, 2008), heme biosynthesis proteins, and DNA transcription and replication-related factors (Scarpulla, 2002; Kelly and Scarpulla, 2004). NRF1 regulates the transcription of mitochondrial transcription factor A (TFAM), a crucial transcription factor that regulates mitochondrial DNA (mtDNA) replication (Virbasius et al., 1993; Virbasius and Scarpulla, 1994). The massive synthesis of mtDNA is necessary for macrophages to form the NLR family pyrin domain-containing 3 (NLRP3) inflammasome, which cleaves mature interleukin-1β (IL-1β) by activating caspase-1 (Zhong et al., 2018). Besides, our previous studies showed that NRF1 transcriptionally activates the p65 subunit of nuclear factor kappa B (NF-κB), thus up-regulating the NF-κB signaling in chronic obstructive pulmonary disease (Wang et al., 2021). However, the activation of NF-κB inhibits the formation of inflammasome (Zhong et al., 2016). Therefore, we speculate that NRF1 plays a complex regulatory role in the activation of microglia under high-altitude exposure, which is worth studying.

The target genes of NRF1 were screened out by chromatin immunoprecipitation (ChIP)–seq technique, and it was found that serious genes were enriched in the endocytosis pathway. The increased expression of phagocytic proteins contributed to microglial activation (Niesman et al., 2013; Portugal et al., 2017; Heckmann et al., 2019). Caveolin 1 (CAV-1) and the adaptor-related protein complex 2 subunit beta (AP2B1), which are necessary participants in caveolin- and clathrin-dependent endocytosis, respectively, were explored (Pearse and Robinson, 1990; Kelly et al., 2014). Thus, we speculate that NRF1 might have a role in the regulation of phagocytosis.

We hypothesized that hypoxia up-regulates NRF1 in microglia, induces inflammatory response, and enhances phagocytic function through transcriptional activation of core target genes, thereby inducing microglia over-activation and leading to HACE. This research focused on the biochemical mechanism of HACE mediated by hyperactivated microglia via the transcription factor NRF1. The molecular mechanisms that NRF1 in microglia induces vasogenic cerebral edema through evoking phagocytosis and damaging BBB, as well as cytotoxic cerebral edema through releasing pro-inflammatory factors and stimulating astrocyte swelling, were also studied. Importantly, the molecular mechanism of transcriptional regulation of NRF1 on CAV-1 and AP2B1 and the role of NRF1 in the regulation of phagocytosis were explored for the first time in this study.

## Results

### Microglial activation accelerates HACE

**Figure 1:**
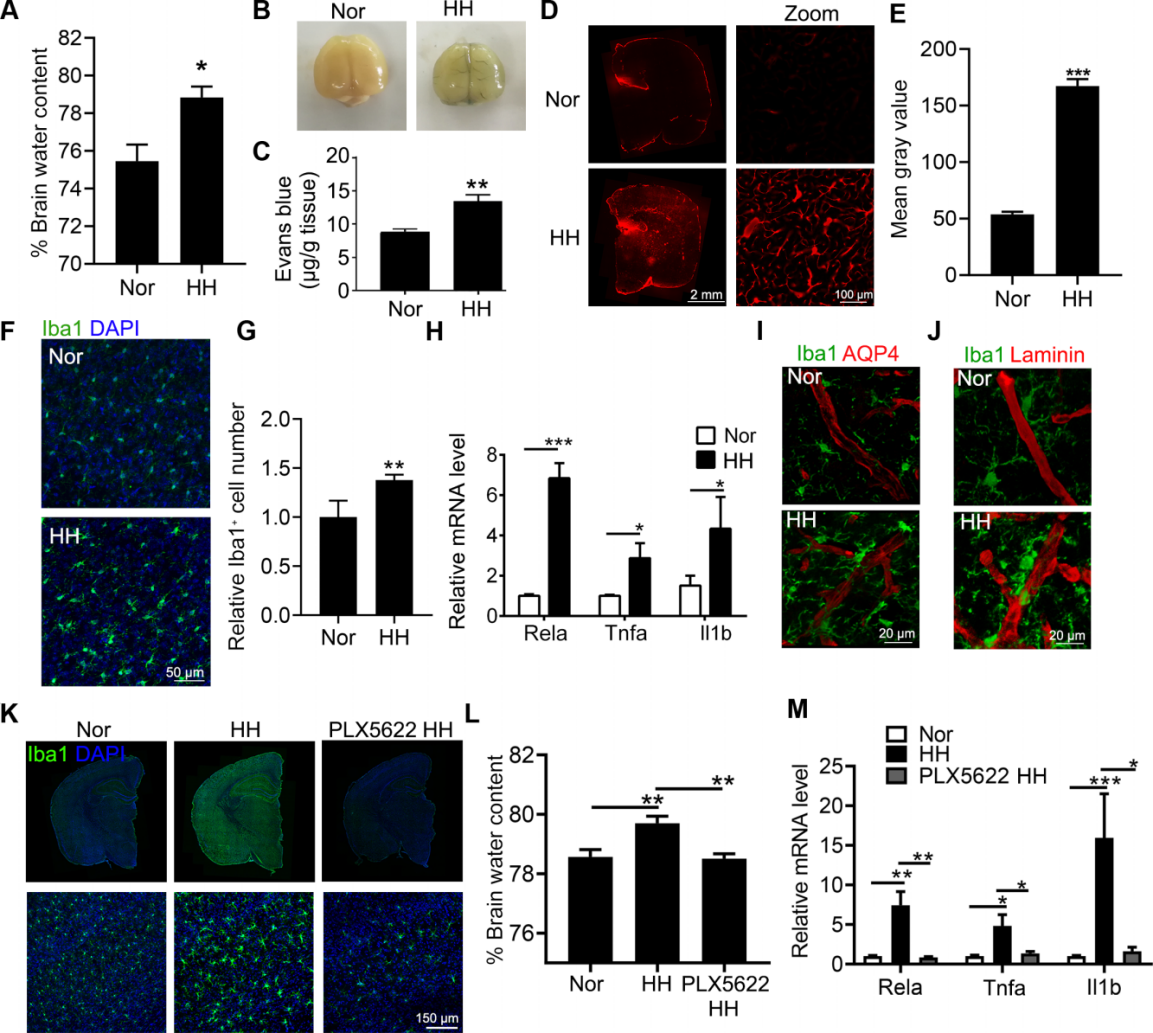
HH induces microglial activation triggering brain edema. C57BL/6J mice were exposed to HH (7000 m above sea level) for 48 h. (**A**) Brain water content was measured by the wet–dry weight method. (**B**–**E**) Mice were injected with Evens blue via tail vein for 1 h and subsequently perfused with normal saline to remove the dye from the circulation. (**B**) The whole brain was harvested to observe Evans blue infiltrated in blood vessels and tissues. (**C**) One half brain was homogenized to quantified dye content by spectrophotometric method. (**D** and **E**) Another half brain was sectioned for confocal imaging at 647 nm laser (**D**) and quantified by Image J software (**E**). (**F** and **G**) Brain sections were incubated with anti-Iba1 antibody to label microglia (**F**) and evaluate cell activation by counting fluorescence intensity (**G**). (**H**) mRNA levels of pro-inflammatory factors *Rela, Tnfa*, and *Il1b* in the cortex tissue were measured by real-time PCR. (**I** and **J**) Brain sections were co-incubated with anti-Iba1 antibody and anti-AQP4 (**I**) or anti-Laminin antibody (**J**) to label microglia and vessel to observe the vascular localization of microglia. (**K**–**M**) C57BL/6J mice were pretreated with PLX5622 followed by exposure to HH. (**K**) Brain sections were incubated with anti-Iba1 antibody to label microglia. (**L**) Brain water content was measured by the wet–dry weight method. (**M**) mRNA levels of pro-inflammatory factors *Rela, Tnfa*, and *Il1b* in the cortex tissue were measured by real-time PCR. *n* = 5, *P < 0.05, **P < 0.01, and ***P < 0.001 by Student's *t*-test.

C57BL/6J mice were exposed to HH (7000 m above sea level) for 48 h to induce HACE. Water content was significantly increased in HH-treated mice, as seen in Figure 1A. To verify the permeability of BBB, mice were injected with Evans blue. The dye residues in the HH-treated mouse brain were visible in Figure 1B. Statistical results also confirmed that Evans blue remained more in HH brains (Figure 1C). Furthermore, the diffusion of dye in brain blood vessels was observed. Figure 1D and E demonstrated significantly stronger signals in both blood vessels and nearby tissues in HH brain sections. All these results suggested that HH exposure leads to increased cerebral vascular permeability. To verify the microglial activation in HH mice, we stained brain sections with Iba1, a microglia/macrophage-specific protein. Figure 1F and G showed an increase in microglial cell bodies, a decrease in branches, and an up-regulation of Iba1^+^ cells in the HH-exposed mouse brain. In consistent, mRNA levels of p65, tumor necrosis factor-alpha (TNF-α), and IL-1β were significantly up-regulated in brain tissues of HH mice (Figure 1H). We further co-stained microglia and vessels with Iba1 and AQP4 or Laminin antibodies. Figure 1I and J showed that activated microglia accumulated to the vessels after HH exposure (Supplementary Videos S1 and S2), indicating that the increased BBB permeability was associated with microglial activation.

PLX5622 is a brain-penetrant colony-stimulating factor 1 receptor inhibitor, which was reported to specifically eliminate microglia (Spangenberg et al., 2019). To clarify whether HACE was related to microglial activation, we fed mice with a PLX5622 diet to deplete microglia. As shown in Figure 1K, Iba1-marked microglia decreased in brain sections of PLX5622-treated mice. When compared to the HH group, microglia depletion significantly reduced brain water content (Figure 1L), suggesting that microglia depletion effectively inhibited HH-induced brain edema. Pro-inflammatory factors including *Rela, Tnfa*, and *Il1b* decreased synchronously after PLX5622 treatment (Figure 1M), further confirming that microglia accelerated cerebral neuroinflammation. In summary, HH-induced brain edema was dependent on activated microglia by enhancing BBB permeability and neuroinflammation.

### Hypoxia stimulates pro-inflammatory microglial activation thus inducing astrocyte swelling through AQP4

**Figure 2:**
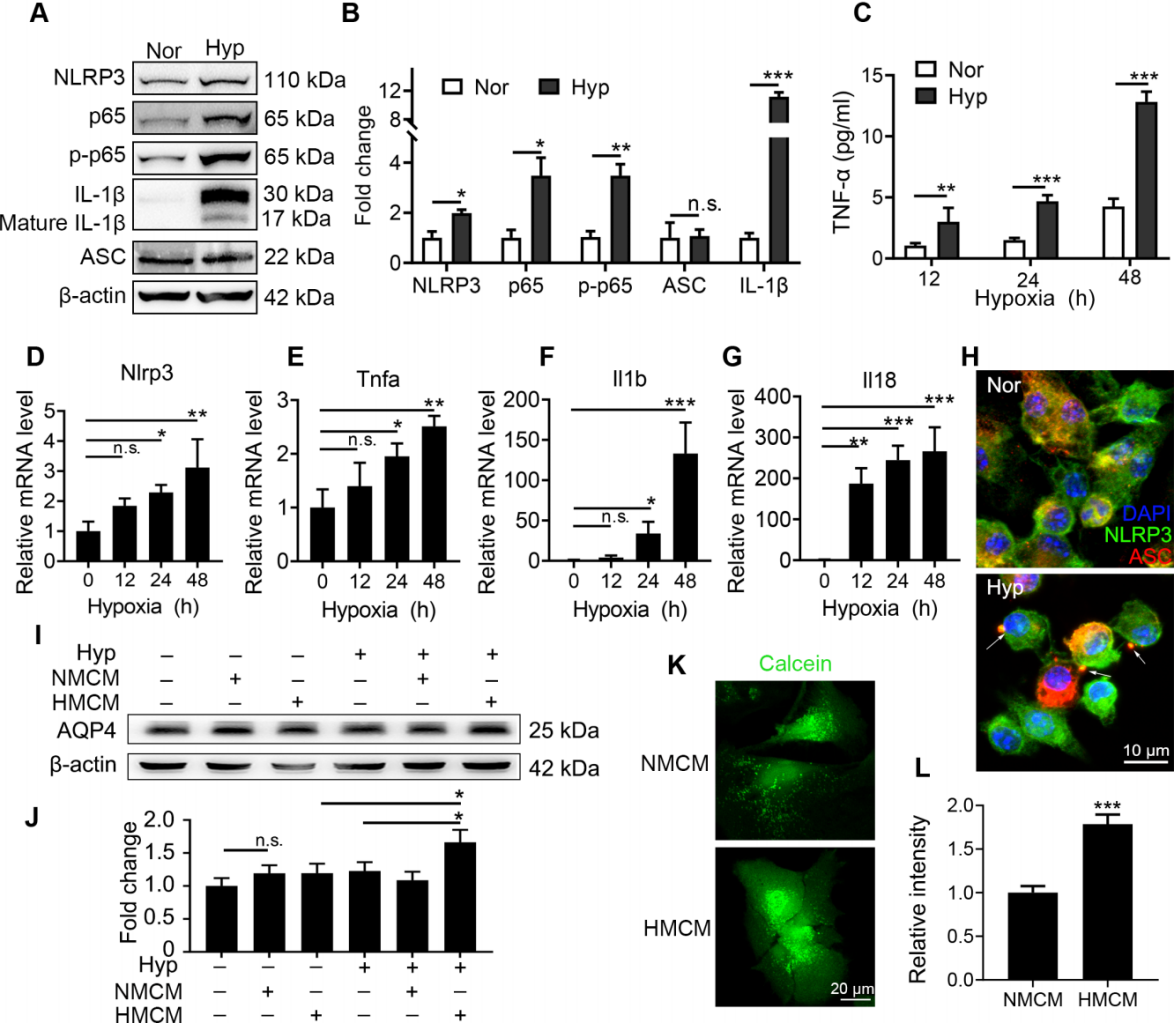
Hypoxia-activated microglia release pro-inflammatory cytokines and induce astrocyte swelling. Primary microglia were exposed to 1% O_2_ for 24 h. (**A** and **B**) Protein levels of NLRP3, p65, p-p65, IL-1β, p-IL-1β, and ASC were determined by western blotting (**A**) and quantified by strip grayscale (**B**, *P < 0.05 and ***P < 0.001 by Student's *t*-test). (**C**) Secretion of TNF-α by microglia to the supernatant of culture medium was measured by ELISA (**P < 0.01 and ***P < 0.001 by Student's *t*-test). (**D**–**G**) mRNA levels of *Nlrp3, Tnfa, Il1b*, and *Il18* were determined by real-time PCR (*P < 0.05, **P < 0.01, and ***P < 0.001 by one-way ANOVA). (**H**) Cells were fixed and stained with NLRP3 and ASC antibodies to display NLRP3 inflammasomes (white arrows). (**I**–**L**) Microglia culture medium was harvested and diluted with the same volume of fresh medium and then incubated with astrocytes under hypoxia. The protein level of AQP4 in astrocytes was measured by western blotting (**I**) and quantified by strip grayscale (**J**, *P < 0.05 by Student's *t*-test). Astrocytes were stained with Calcein green AM (**K**) and the astrocyte swelling status induced by microglia medium was quantified by the dye intensity (**L**, ***P < 0.001 by Student's *t*-test). n.s. indicates no significance.

To verify the effect of hypoxia on microglial activation *in vitro*, primary microglia were exposed to 1% O_2_ for 12, 24, or 48 h. Hypoxia promoted p65 phosphorylation and up-regulated NF-κB downstream targets IL-1β and NLRP3 in the microglia with 24 h hypoxia (Figure 2A and B), indicating that hypoxia stimulated NF-κB signaling in microglia. TNF-α level in culture medium, as measured by enzyme-linked immunosorbent assay (ELISA), increased significantly with prolonged duration of hypoxia (Figure 2C). Consistently, mRNA levels of pro-inflammatory factors including NLRP3, TNF-α, IL-1β, and IL-18 were also up-regulated time-dependently on hypoxia (Figure 2D–G). Furthermore, NLRP3 inflammasomes were observed in primary microglia with 24 h hypoxia exposure by co-staining NLRP3 and apoptosis-associated speck-like protein containing a C-terminal caspase recruitment domain (ASC) (Figure 2H). Based on these results, hypoxia induced microglial activation and promoted pro-inflammatory factor release. Next, we investigated whether the cytokines secreted by hypoxia-induced microglia promoted astrocyte swelling. Normal or hypoxic microglia culture medium was diluted with the same volume of fresh DF-12 medium as NMCM or HMCM, respectively. Primary astrocytes were cultured with NMCM or HMCM and exposed to 1% O_2_ for 24 h. Figure 2I and J showed that AQP4, the main aquaporins in astrocytes, was only significantly up-regulated in both HMCM and hypoxia-treated astrocytes. Besides, astrocyte swelling assay was performed by labelling NMCM- or HMCM-treated hypoxic astrocytes with Calcein green AM. The intensity of Calcein green AM significantly increased in HMCM-treated hypoxic astrocytes (Figure 2K and L), demonstrating that hypoxia-associated astrocyte swelling was dependent on activated microglia.

### NRF1 is associated with hypoxia-activated microglia both in vivo and in vitro

**Figure 3:**
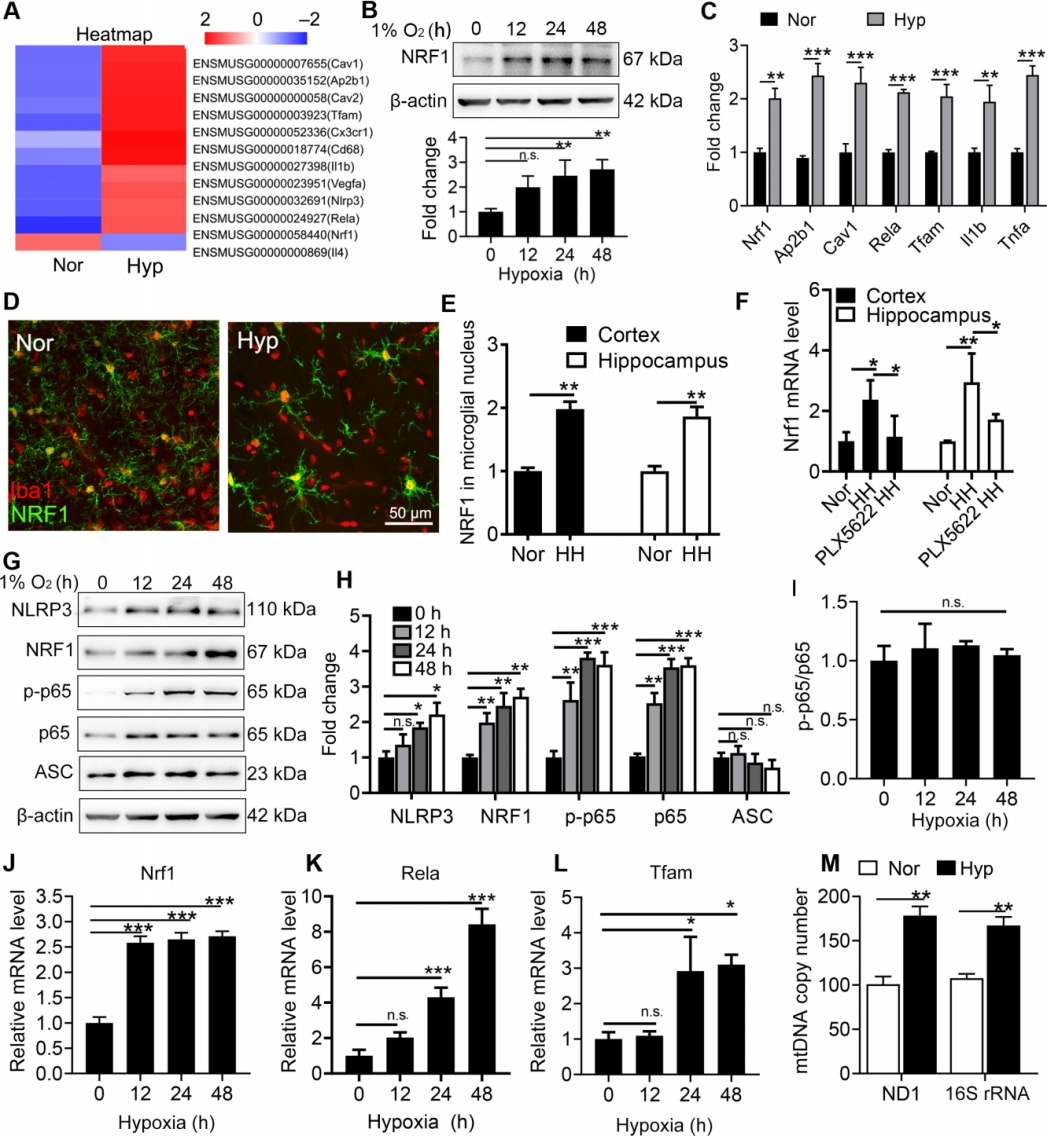
Hypoxia up-regulates NRF1 both *in vivo* and *in vitro*. (**A**) Primary microglia were treated with hypoxia for 24 h followed by RNA-seq analysis. Differentially expressed genes were displayed by Heatmap. (**B**) Primary microglia were incubated under hypoxia for the indicated duration. The protein level of NRF1 was measured by western blotting and quantified (**P < 0.01 by one-way ANOVA). (**C**–**F**) Adult CX3CR1^GFP/+^ mice were exposed to HH or pretreated with PLX5622 followed by exposure to HH. (**C**) Microglia were isolated by FACS. mRNA levels of *Nrf1*, Ap2*b1, Cav1, Rela, Tfam, Il1b*, and *Tnfa* were measured by real-time PCR (**P < 0.01 and ***P < 0.001 by Student's *t*-test). (**D**) Brain sections were stained with anti-Iba1 and anti-NRF1 antibodies to display the distribution pattern of NRF1 in microglia. (**E**) NRF1 signal in the nuclei of microglia was quantified (**P < 0.01 by Student's *t*-test). (**F**) The mRNA level of Nrf1 in brain tissues was measured by real-time PCR (*P < 0.05 by Student's *t*-test). (**G**–**L**) Primary microglia were incubated under hypoxia for the indicated duration. Protein and mRNA levels of NRF1 and inflammatory factors were measured by western blotting (**G**–**I**) and real-time PCR (**J**–**L**). *P < 0.05, **P < 0.01, and ***P < 0.001 by one-way ANOVA. (**M**) mtDNA copies in microglia exposed to hypoxia for 24 h were quantified by real-time PCR (**P < 0.01 by Student's *t*-test). n.s. indicates no significance.

RNA-seq of hypoxia-treated microglia was performed to clarify the mechanism of microglial activation by hypoxia. A group of genes were up-regulated after hypoxia treatment (Figure 3A). Interestingly, both *Nrf1* and its target gene *Tfam* increased in hypoxic microglia. This was confirmed in Figure 3B that NRF1 protein level increased significantly with the prolonged duration of hypoxia. In order to study the changes of microglia *in vivo*, we exposed CX3CR1^GFP/+^ mice to HH. CX3CR1^GFP/+^ mice express EGFP in dendritic cells and brain microglia under control of the endogenous *Cx3cr1* locus, and thus are useful in the studies of microglial migration and trafficking (Wang et al., 2020). We isolated microglia from HH-treated adult CX3CR1^GFP/+^ mouse brain by fluorescence-activated cell sorting (FACS). In consistent with *in vitro* results, expression levels of *Nrf1, Ap2b1, Cav1, Rela, Tfam, Il1b*, and *Tnfa* were up-regulated after HH treatment (Figure 3C). The NRF1 level was also up-regulated in brain tissues from mice exposed to HH. Figure 3D and E proved that nuclear NRF1 was up-regulated in microglia of both cortex and hippocampus after mice were exposed to HH. Moreover, depletion of microglia by PLX5622 significantly reduced the *Nrf1* level in both cortex and hippocampus after HH exposure (Figure 3F). Since NRF1 protein level showed no significant changes in primary neurons, astrocytes, or bEnd.3 cells (Supplementary Figure S2), we concluded that hypoxia up-regulated microglial NRF1 both *in vivo* and *in vitro*. Subsequently, the correlation between NRF1 and inflammatory factors in microglia under persistent hypoxia was investigated. NRF1, p65, and NLPR3 were up-regulated synchronously in a time-dependent manner upon hypoxia (Figure 3G and H). Interestingly, hypoxia did not affect the ratio of p-p65 to p65 (Figure 3I), suggesting that the phosphorylation of p65 induced by hypoxia was based on an increase in p65. Moreover, mRNA level of *Tfam*, the target gene of NRF1, and mtDNA copy number were also up-regulated in consistent with *Nrf1* and *Rela* (Figure 3J–M), indicating the activation of the NRF1 pathway in hypoxic microglia.

### NRF1 transcriptionally regulates NF-κB and TFAM thus promoting pro-inflammatory responses in hypoxic microglia

**Figure 4:**
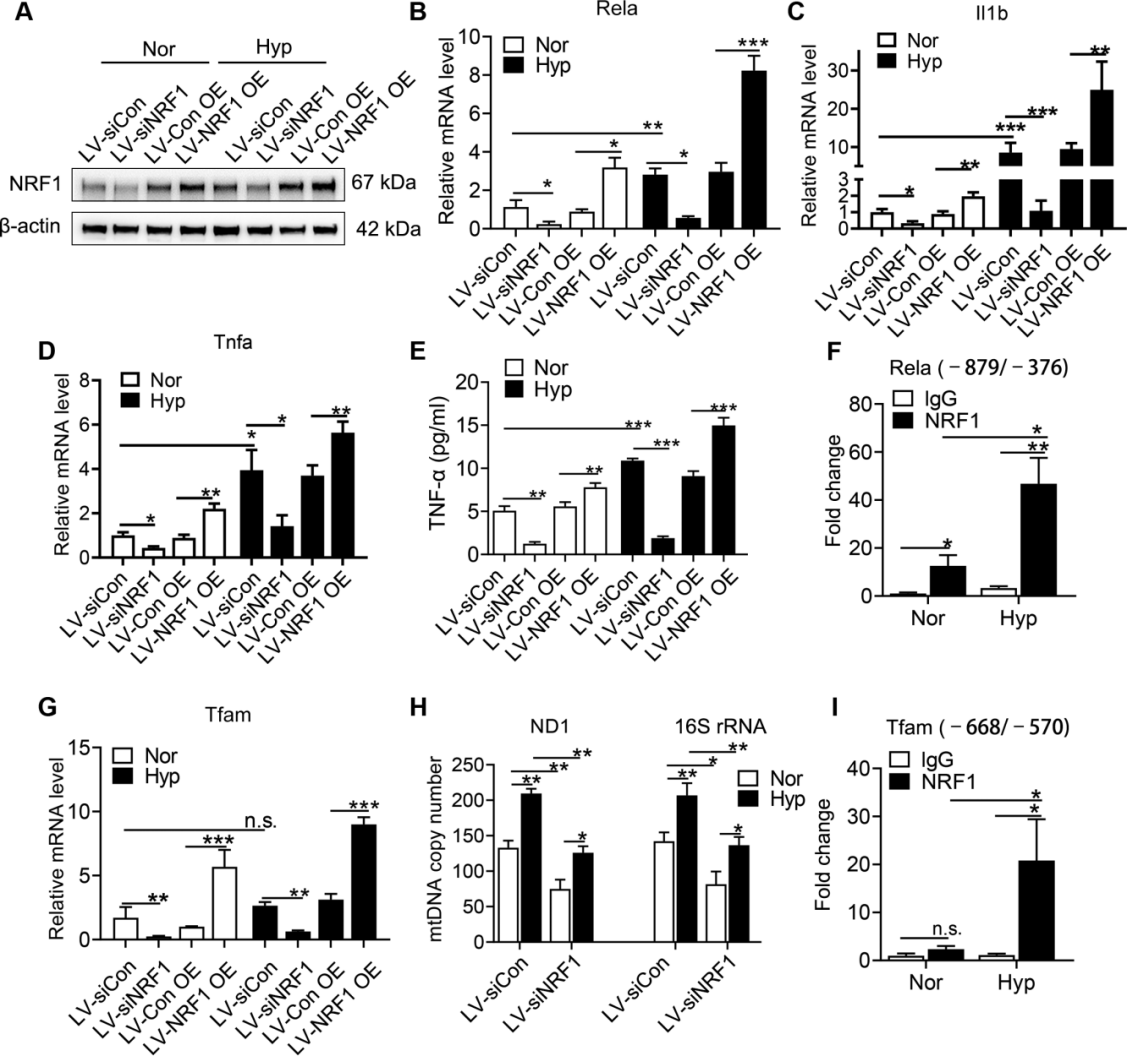
Transcriptional regulation of NRF1 on NF-κB and TFAM is up-regulated under hypoxia. Primary microglia were infected with lentivirus followed by hypoxia treatment for 24 h. (**A**) The protein level of NRF1 was measured by western blotting. (**B**–**D**) mRNA levels of *Rela, Il1b*, and *Tnfa* were determined by real-time PCR (*P < 0.05, **P < 0.01, and ***P < 0.001 by two-way ANOVA). (**E**) TNF-α secretion by microglia to the supernatant of culture medium was measured by ELISA (**P < 0.01 and ***P < 0.001 by two-way ANOVA). (**F**) Enrichments of the *Rela* promoter by NRF1 were measured by ChIP–qPCR (*P < 0.05 and **P < 0.01 by Student's *t*-test). (**G** and **H**) The mRNA level of *Tfam* (**G**) and mtDNA copies (**H**) were determined by real-time PCR (*P < 0.05, **P < 0.01, and ***P < 0.001 by two-way ANOVA). (**I**) Enrichments of the *Tfam* promoter by NRF1 were measured by ChIP–qPCR (*P < 0.05 by Student's *t*-test). n.s. indicates no significance.

To verify the function of NRF1 in microglial activation, we modulated NRF1 levels by lentivirus infection. As shown in Figure 4A, the protein level of NRF1 was down-regulated in microglia infected by lentivirus expressing NRF1 siRNA (siNRF1) whereas up-regulated in NRF1-overexpressing (NRF1 OE) microglia under both normoxia and hypoxia. *Rela, Il1b*, and *Tnfa* were also down-regulated or up-regulated synchronously under both normoxia and hypoxia conditions (Figure 4B–D). In consistent, TNF-α secretion was also regulated by NRF1 (Figure 4E), showing that NRF1 mediated hypoxia-induced pro-inflammatory microglia. ChIP–quantitative polymerase chain reaction (qPCR) was carried out to determine whether p65 was transcriptionally regulated by NRF1. NRF1 enriched the promoter region of *Rela*, which showed a higher enrichment fold under hypoxia (Figure 4F). The inhibition of p65 by Bay-11 7082 reduced both p65 and IL-1β instead of NRF1 (Supplementary Figure S3), supporting that NRF1 induced inflammation dependent on transcriptional regulation of p65. *Tfam*, as a target gene of NRF1, showed synchronous trends in mRNA level (Figure 4G), leading to mtDNA changes in microglia (Figure 4H). Furthermore, NRF1 transcriptionally activated *Tfam* by directly binding to its promoter region (Figure 4I), indicating that NRF1 participated in hypoxia-induced inflammasome by up-regulating mtDNA copies via TFAM transcriptional activation.

### NRF1 mediates hypoxia-enhanced microglia phagocytosis to break the tight junction of endothelial cells

**Figure 5:**
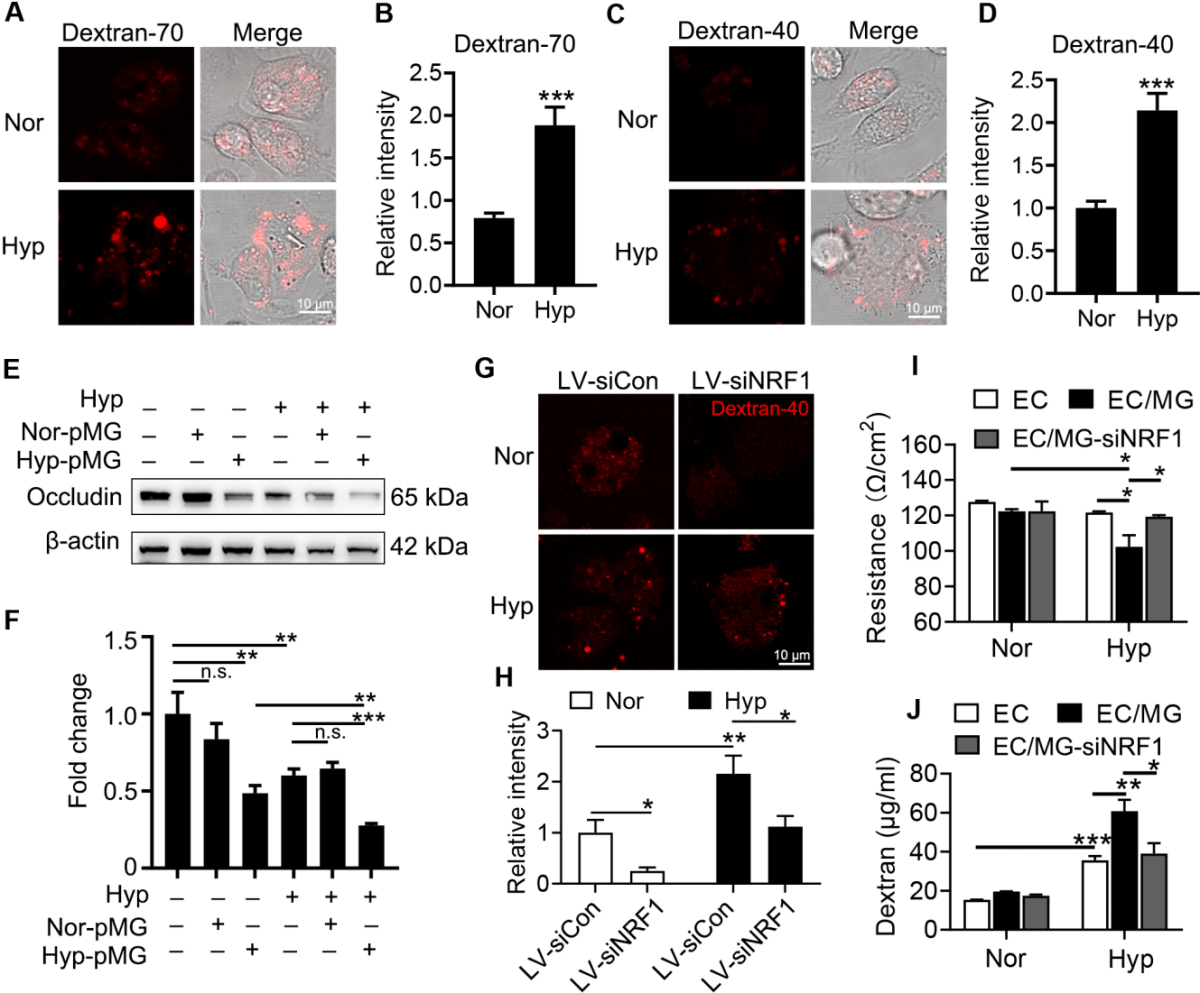
Hypoxia enhances the internalization of microglia dependent on the up-regulation of NRF1. (**A**–**D**) Primary microglia after 24 h hypoxia were incubated with Texas red-labelled Dextran-70 (**A** and **B**) and Dextran-40 (**C** and **D**) for 30 min to determine clathrin-dependent and caveolin-dependent endocytosis, respectively (***P < 0.001 by Student's *t*-test). (**E**–**J**) Hypoxia-pretreated microglia were co-cultured with bEnd.3 followed by hypoxia treatment for 24 h. (**E** and **F**) The protein level of Occludin in bEnd.3 cells was measured by western blotting and quantified (**P < 0.01 and ***P < 0.001 by Student's *t*-test). (**G** and **H**) Hypoxic microglia with normal or low level of NRF1 were incubated with Texas red-labelled Dextran-40 for internalization measurement (*P < 0.05 and **P < 0.01 by two-way ANOVA). (**I** and **J**) Tight junction (**I**) and permeability (**J**) of endothelial cells were measured by resistance or dextran transcytosis, respectively, in the co-culture of bEnd.3 cells (EC) and microglia with normal (MG) or low level of NRF1 (MG-siNRF1) (*P < 0.05, **P < 0.01, and ***P < 0.001 by Student's *t*-test). n.s. indicates no significance.

It has been reported that hypoxia-activated microglia are characterized by up-regulation of phagocytosis (Jolivel et al., 2015; Bok et al., 2017; Iannucci et al., 2022). The hypoxia-treated microglia were incubated with Texas red-labelled 70000 MW dextran (Dextran-70) or 40000 MW dextran (Dextran-40) to determine the clathrin-dependent or caveolin-dependent phagocytosis, respectively. Hypoxia treatment up-regulated clathrin-dependent and caveolin-dependent endocytosis (Figure 5A–D). Then, whether hypoxia-activated microglia affected the tight junction of endothelial cells was further investigated. bEnd.3 cells were co-cultured with hypoxia-pretreated microglia followed by hypoxia for 24 h. Occludin, a tight junction protein, was found significantly reduced in normal bEnd.3 cells co-cultured with hypoxic microglia. Besides, Occludin in bEnd.3 cells was directly down-regulated under hypoxia, and decreased further after co-culture with hypoxic microglia (Figure 5E and F), suggesting that both hypoxia condition and activated microglia injured the tight junction of endothelium and had a superposition effect. The microglia were then infected with siNRF1 lentivirus to investigate whether NRF1 was involved in hypoxia-enhanced phagocytosis. Figure 5G and H showed that siNRF1 groups had lower Dextran-40 signal under both normoxia and hypoxia, suggesting that hypoxia-enhanced phagocytosis was regulated by NRF1 up-regulation. The tight junction of endothelial cells was further investigated by transepithelial electrical resistance (TEER) and transcytosis assay (Li et al., 2020). The interference of NRF1 rescued the damage of tight junction as well as the dextran leakage induced by activated microglia (Figure 5I and J). These results indicated that NRF1 mediated the phagocytosis of hypoxia-enhanced microglia and the injury of tight junction between endothelial cells.

### NRF1 up-regulates microglia phagocytosis of microglia through transcriptional activation of AP2B1 and CAV-1

**Figure 6:**
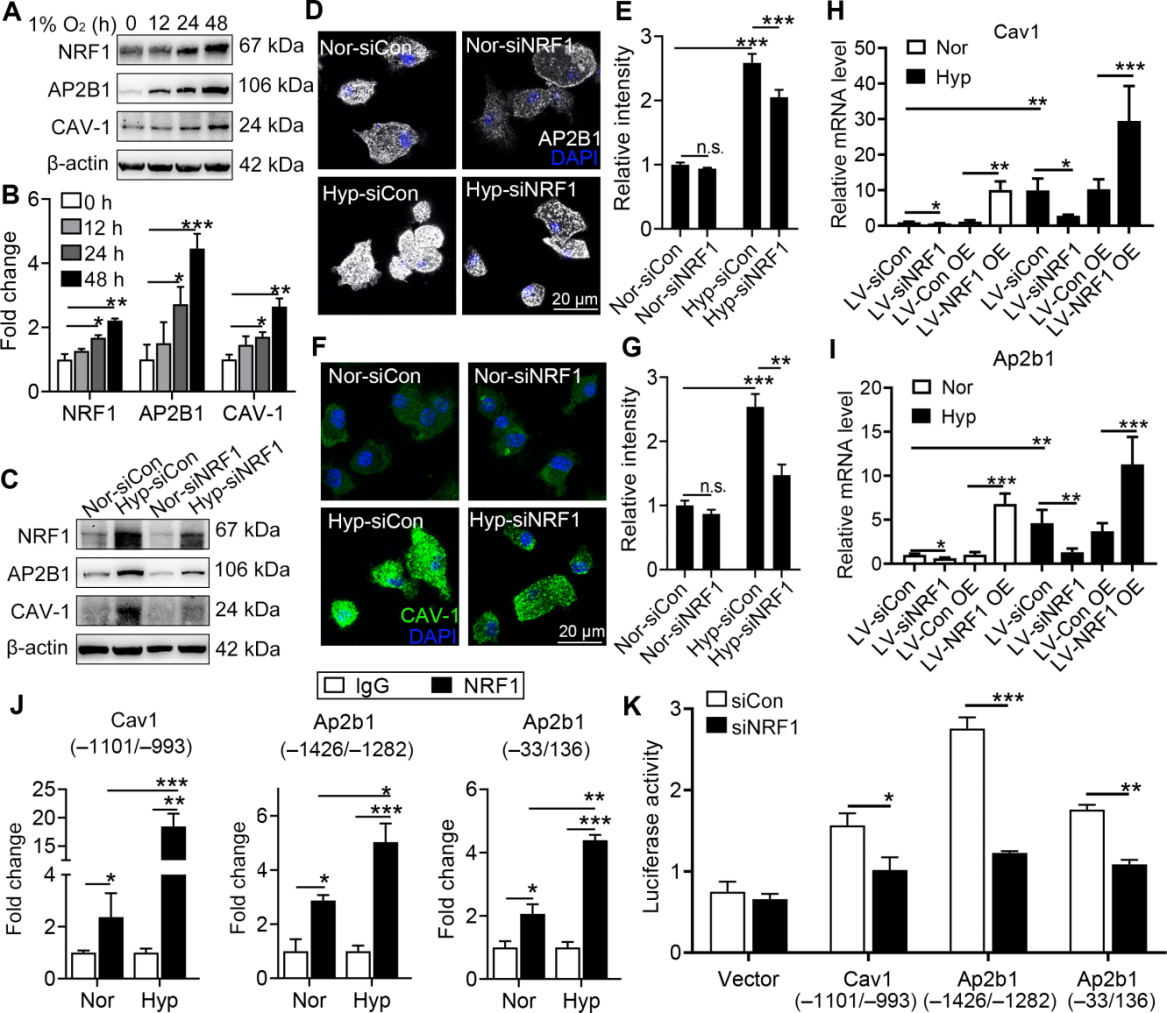
NRF1 transcriptionally regulates AP2B1 and CAV-1 to enhance microglia phagocytosis. (**A** and **B**) Primary microglia were incubated under hypoxia for the indicated duration. Protein levels of NRF1, AP2B1, and CAV-1 were measured by western blotting and quantified (*P < 0.05, **P < 0.01, and ***P < 0.001 by one-way ANOVA). (**C**–**G**) Microglia were transfected with lentivirus expressing siNRF1 and exposed to hypoxia. (**C**) Protein levels of AP2B1 and CAV-1 were determined by western blotting. (**D**–**G**) Cells were fixed and stained with anti-AP2B1 (**D** and **E**) and anti-CAV-1 antibodies (**F** and **G**) (**P < 0.01 and ***P < 0.001 by two-way ANOVA). (**H** and **I**) mRNA levels of *Cav1* and *Ap2b1* in microglia with NRF1 silencing or overexpression after hypoxia were measured by real-time PCR (*P < 0.05, **P < 0.01, and ***P < 0.001 by two-way ANOVA). (**J**) Enrichments of the *Cav1* and *Ap2b1* promoters by NRF1 were measured by ChIP–qPCR (*P < 0.05, **P < 0.01, and ***P < 0.001 by Student's *t*-test). (**K**) Transcriptional activities of the *Cav1* and *Ap2b1* promoters in microglia with normal or low level of NRF1 were determined by dual-luciferase reporter assay (*P < 0.05, **P < 0.01, and ***P < 0.001 by Student's *t*-test). n.s. indicates no significance.

To investigate the mechanism of NRF1-regulated microglia phagocytosis, ChIP–seq followed with Kyoto Encyclopedia of Genes and Genomes pathway analysis was carried out. It was found that NRF1 potentially regulated several genes related to endocytosis, including *Dnm1, Ap2b1, Cav1*, and *Smurf2* (Supplementary Figure S4). AP2B1 and CAV-1 increased synchronously with NRF1 in a time-dependent manner in response to hypoxia (Figure 6A and B). The interference of NRF1 resulted in a decrease of both AP2B1 and CAV-1 under both normoxia and hypoxia (Figure 6C–G). In consistent, *Cav1* and *Ap2b1* were also down-regulated or up-regulated in siNRF1 or NRF1 OE microglia, respectively, under normoxia and hypoxia (Figure 6H and I), showing that NRF1 mediated hypoxia-promoted phagocytosis in microglia. According to ChIP–qPCR results in Figure 6J, NRF1 enriched the promoter regions of *Cav1 (–1101/–993), Ap2b1 (–1426/–1282)*, and *Ap2b1 (–33/136)*, with higher enrichment folds during hypoxia. Thus, dual-luciferase reporter assays were carried out to detect the transcriptional regulation of NRF1 on the target genes. The transitional activities of *Cav1* and *Ap2b1* reporter genes were down-regulated in HEK293T cells transfected with siNRF1 (Figure 6K), showing that NRF1 positively regulated *Cav1* and *Ap2b1* transcription and thus triggered microglia phagocytosis.

## Discussion

This study explored the mechanism of microglial activation by hypoxia and the pathological effect of activated microglia on HACE. The major experimental findings include (i) microglia are obviously activated and migrate to cerebral microvessels in HACE mice; (ii) depletion of microglia significantly reduces the occurrence of HACE; (iii) 1% O_2_ promotes microglia releasing cytokines, and pro-inflammatory microglial activation induces astrocyte swelling; (iv) hypoxia enhances endocytosis mediated by clathrin and caveolin in microglia; (v) hypoxia-up-regulated NRF1 accelerates pro-inflammatory response through transcriptional activation of NF-κB p65 and TFAM; and (vi) NRF1 binds to and activates the transcription of *Cav1* and *Ap2b1*, contributing to hypoxia-enhanced phagocytosis in microglia. All results support our hypothesis, indicating the effects of NRF1-mediated microglial activation on the pathology of HACE by accelerating pro-inflammatory activation and BBB injury.

Rodents are significantly more tolerant to hypoxia than humans, and thus do not cause AMS at 4500–5000 m altitude exposure. Our findings revealed that rodents did not show signs of HACE even at 6000 m altitude exposure (data not shown). Thus, to simulate a HACE model, mice were exposed to 7000 m for 48 h. Zhou et al. (2017) used lipopolysaccharide (LPS) combined with 6000 m altitude exposure to induce HACE. They suggested that the LPS-induced inflammatory response promoted HACE (Zhou et al., 2017). However, our study aimed to investigate the mechanisms by which extreme HH environments induce brain edema. The addition of LPS altered the conditions under which brain edema is induced. It was observed in our study that HACE was induced in mice exposed to 7000 m above sea level for 48 h, and Evens blue imaging results revealed vascular leakage near the corpus callosum, which is consistent with the onset and symptoms of HACE in humans (Hackett, 1999; Urushida et al., 2021).

HACE is characterized by brain edema and high intracranial pressure (Hackett, 1999; Urushida et al., 2021). Magnetic resonance (MR) imaging research in HACE patients suggests edema in the white matter corpus callosum rather than in the gray matter, leading to a hypothesis about the vasogenic origin for the edema (Hackett, 1999). Recent research, however, has found evidence of cytotoxicity in HACE (Kallenberg et al., 2007; Zhou et al., 2017). MR imaging data revealed intracellular cytotoxic edema in combination with an anatomic predisposition to a tight-fit brain, which may prove of pathophysiologic significance (Kallenberg et al., 2007). Systemic inflammation induced by LPS contributes to hypoxia-induced brain edema (Zhou et al., 2017). Thus, both vasogenic edema and cytotoxic edema might be involved in the formation of HACE. Since over-activated microglia are reported to contribute to both cytotoxic edema and vasogenic edema (Tanaka et al., 2018; Chen et al., 2019; Haruwaka et al., 2019; Murata et al., 2020), we asked whether microglia benefit from HACE. In this study, hypoxia-induced activation of microglia, both *in vivo* and *in vitro*, was verified. Depletion of microglia attenuated the brain edema process, proving a key function of microglia in HACE. Furthermore, dexamethasone is mainly used to prevent and treat HACE by reducing vascular permeability and inhibiting inflammation (Swenson, 2016). Considering the main function of microglia on the inflammatory response, it is easy to understand the therapeutic effect of glucocorticoids on serving AMS and HACE. However, the side effects of glucocorticoids should not be overlooked, as it also affects multiple health systems. Therefore, finding clinical strategies to target and inhibit microglial activation is important for the effective prevention and treatment of HACE.

Microglia are the only immune cells present in the central nervous system parenchyma and are thus the first responders to environmental changes (Waisman et al., 2015). Microglia become activated and transform from ramified morphology to amoeboid shape if exposed to hazardous signals such as pro-inflammatory cytokines, injury, and cellular stress (Fernandez-Arjona et al., 2019). Activated microglia are accompanied by the activation of toll-like receptor signal and the increase of phagocytosis (He et al., 2021). However, there is rare evidence that demonstrates the relationship between up-regulation of inflammatory pathway and increase of phagocytosis. Jolivel et al. (2015) has reported that long-term ischemia induced phagocytic phenotype of microglia (Jolivel et al., 2015). Iannucci et al. (2022) showed that both high glucose and hypoxia increased microglia phagocytosis. The known mechanism of the up-regulation of microglia phagocytosis induced by hypoxia may be related to the activation of hypoxia-inducible factor (HIF) pathway (Bok et al., 2017). In the early stages of ischemia, enhanced microglia phagocytosis is beneficial for maintaining cellular homeostasis, cleaning cellular waste, and reducing pro-inflammatory responses. However, persistent ischemia causes excessive activation of microglia, which phagocytizes endothelial cells thus inducing endothelial damage (Haruwaka et al., 2019). Therefore, the regulation of microglia during brain injury is quite complex, and whether microglia phagocytosis is beneficial or detrimental is related to the activation status and environment.

For the first time, we revealed a dual role of NRF1 in mediating inflammatory response and phagocytosis. Because of its importance in mitochondrial regulation and NF-κB signaling, the up-regulation of NRF1 could explain how hypoxia affected both mitochondrial homeostasis and pro-inflammatory responses. Microglia phagocytosis is primarily mediated by TREM2, TALs, and LRPR (Fu et al., 2014). However, phagocytosis requires the assistance of phagocytosis-related proteins, including clathrin-dependent and independent pathways. Clathrin-dependent endocytosis requires the involvement of AP2, while clathrin-independent endocytosis is mainly mediated by CAV-1 (Pearse and Robinson, 1990; Kelly et al., 2014). Therefore, NRF1-mediated up-regulation of AP2B1 and CAV-1 enhances microglia phagocytosis. In conclusion, targeting microglial NRF1 may be a potential strategy for the treatment of HACE.

In 2016, Wang et al. (2016) reported that NRF1 participates in the hypoxic response of cells via transcriptional repression of HIF-1α. Since it is well known that HIFs are involved in hypoxia-induced microglial activation and neuron injury (Huang et al., 2014; Fang et al., 2020; Yuan et al., 2021), we speculated that NRF1 might also activate microglia through up-regulation of HIF signaling. Further studies have been conducted regarding the function of NRF1 in different tissues and organs under hypoxia. Indeed, hypoxia affects NRF1 differently dependent on tissues. Hypoxia represses NRF1 in testicular tissues, affecting testosterone synthesis via transcriptional regulation of steroidogenic acute regulatory protein (Wang et al., 2017, 2019). However, NRF1 is up-regulated in vascular endothelium and has a regulatory effect on hypoxia-induced blood pressure elevation by affecting endothelin 1 and angiotensin-converting enzyme (Jiang et al., 2021). Moreover, in chronic obstructive pulmonary disease, NRF1 was found to mediate inflammatory injury through the regulation of the NF-κB signaling pathway in pulmonary epithelial cells (Jiang et al., 2021). NRF1 expression was found up-regulated in an endotoxin-induced acute lung injury model to mediate mitochondrial biosynthesis (Shi et al., 2019). NRF1 may be a key factor in the development of acute lung injury (Liang et al., 2021). Yang et al. (2018) found that NGLY1 gene-mediated mitochondrial homeostasis and inflammatory responses were dependent on NRF1 up-regulation. This study suggests that NRF1 plays a key regulatory role in cellular response, adaptation, and stress processes. The role of NRF1 in cell fate deserves an in-depth investigation.

In conclusion, this study demonstrates that hypoxia-activated microglia trigger HACE. The underlying molecular mechanism might be traced in part to the transcriptional regulation of NRF1. Hypoxia up-regulates NRF1 in microglia, induces inflammatory response via transcriptional activation of NF-κB p65 and TFAM, and enhances phagocytic activity via CAV-1 and AP2B1. The over-activated microglia subsequently migrate to blood vessels, disrupting the integrity of the BBB. HACE is eventually induced by astrocyte swelling caused by pro-inflammatory factors. These findings suggest that targeted inhibition of microglial activation should be considered as a potential therapeutic approach for HACE.

## Materials and methods

### Ethics approval and consent to participate

All the studies reported here were submitted to the Ethics Committee on Animal Experimentation of Nantong University and all procedures were approved by the Animal Care and Use Committee of Nantong University and the Jiangsu Province Animal Care Ethics Committee (Approval ID: SYXK(SU)2007-0021).

### Microglia depletion and HH treatment

Adult male CX3CR1^GFP/+^ (005582, B6.129P2 (Cg)-Cx3cr1^tm1Litt^/J) supplied from Jackson Laboratory were a gift from Dr Xia Li at Nantong University. Adult male C57BL/6J mice (8 weeks old, 20–23 g) were provided by the Experimental Animal Center of Nantong University and were accommodated for one week before experiments with a 12 h light–dark cycle. For PLX5622-induced microglial depletion, mice were placed on a chow diet with PLX5622 incorporated at 1200 ppm (Willis et al., 2020; Enlow et al., 2021). Control mice received standard mouse chow. For HH treatment, mice were kept in a 60 cm × 40 cm × 40 cm chamber, which can simulate different altitude. Mice rose to 7000 m altitude from sea level at a speed of 5 m/sec and stayed for 48 h. Then, the mice descended to sea level at a speed of 5 m/sec. Finally, 30 min after the HH treatment, the mice were euthanized and perfused with phosphate-buffered saline solution via the left ventricle to remove the blood.

### Brain water content assay

Brain water content was measured by the wet–dry weight method (Bauer et al., 2021). Samples of the whole-brain cortex were placed on small silver paper that was weighed before usage and in the following step together with the samples to calculate the wet weight. The cardboards containing the samples were then incubated in a drying oven at a constant temperature of 900°C and weighed several times to get a stable weight as dry weight. The brain water content was then calculated as follows: brain water content = (wet weight—dry weight)/weight × 100%.

### BBB permeability assay

BBB permeability assay was performed as previously described (Goldim et al., 2019). Evans blue dye (2% *w*/*v*) was administered intravenously via the tail vein and allowed to circulate for 1 h before being removed through intracardial perfusion with normal saline. One half brain was homogenized and residual content of the dye in the tissue was measured by spectrophotometric method. Another half brain was fixed and sectioned for confocal imaging at 647 nm laser by Leica SP8.

### FACS

After HH treatment, CX3CR1^GFP/+^ mice were immediately euthanized and decapitated. Brains were quickly dissected and removed to ice-cold D-Hank's buffer. Then, single cell suspension was prepared according to a Papain-based single cell isolation method (Yousef et al., 2018). CX3CR1^+^ microglia were purified by FACS. In average 2 × 10^5^ cells were isolated from a mouse brain. The isolated cells were further lysed for real-time PCR.

### Primary astrocyte and microglia culture

As previously described (Wu et al., 2013, 2017; Wong et al., 2020), primary neonatal astrocytes and microglia were prepared from cerebral cortices of 1-day old neonatal mice. After removal of the meninges, cortical tissue was digested by trypsin. Then, cells were cultured in Dulbecco's modified Eagle's medium F12 (DF-12) supplemented with 10% fetal bovine serum and penicillin/streptomycin (100 U/ml and 100 mg/ml, respectively) at 37°C in a 5% CO_2_ humidified incubator. After 10 days of growth, primary astrocytes were characterized by GFAP antibody (Supplementary Figure S1A). For microglia collection, 5 ng/ml granulocyte–macrophage colony-stimulating factor (78017, STEMCELL) were supplemented in the culture medium of primary astrocytes for 3 days. Cells from the supernatant were then harvested and seeded in 12-well plates. Immunostaining of Iba1 was used for the characterization of microglia (Supplementary Figure S1B).

### Astrocyte swelling measurement

Primary microglia were treated with 1% O_2_ for 24 h. Culture medium was collected and spun at 1000× *g* to remove floating microglia. The medium produced by normal or hypoxia-treated microglia was diluted twice by mixing it with the same volume of fresh DF-12 medium, as NMCM or HMCM, respectively. Primary astrocytes labelled with Calcein green AM were incubated by NMCM or HMCM and exposed to 1% O_2_ for 24 h. Finally, astrocytes were imaged in real time at 488 nm for swelling assay. The intensity of Calcein green AM is related to the swelling condition.

### Lentivirus infection

Primary microglia were harvested, counted, and immediately mixed with lentivirus at MOI = 1, and then were seeded on 12-well plates for 48 h followed by hypoxia treatment. The efficacy of infection was ∼70% by detecting GFP signal under microscope.

### Phagocytosis assay

Primary microglia were incubated with 100 μg/ml Texas red-labelled Dextran-70 (D1830, Thermo) or Dextran-40 (D1829, Thermo) for 15 min. Then the cells were chased with fresh medium immediately for confocal imaging at 552 nm by Leica SP8.

### TEER and transcytosis assays

bEnd.3 cells were seeded on 12-transwell inserts (3401, Corning) and cultured to reach 90% confluence. Primary microglia were seeded on top of endothelial cells and co-cultured for 24 h. For the TEER assay, cells were incubated with D-Hank's buffer for 30 min. The total resistance of the co-culture cells was measured by the Millicell ERS-2 Epithelial Volt-ohm Meter (Merck Millipore). Resistance of HBSS was recorded as blank resistance. TEER was calculated using the following formula: TEER (Ω × cm^2^) = (total resistance − blank resistance) (Ω) × insert area (cm^2^). Lower resistance indicated damage to the tight junction. For transcytosis assay, 250 μg/ml Dextran-40 was added in inserts for 1 h. The medium in the lower plate was then collected and measured with a spectrophotometer to determine the concentration of dextran.

### Western blotting

Cells and tissues were lysed with RIPA buffer, and protein concentration was calculated by bicinchoninic acid assay. Proteins were isolated by sodium dodecyl sulfate–polyacrylamide gel electrophoresis and transferred to polyvinylidene fluoride membranes. Membranes were blocked with 5% non-fat dry milk for 1 h at room temperature, and then incubated with primary antibodies including anti-AQP4 (Cell Signaling Technology, 59678S), anti-ASC (Cell Signaling Technology, 60824S), anti-AP2B1 (Santa Cruz, 74423), anti-CAV-1 (Santa Cruz, sc-53564), anti-NLRP3 (Life Science, mAG-20B-0014), anti-NRF1 (Abcam, ab175932), anti-Occludin (Proteintech, 66378), anti-p65 (Cell Signaling Technology, 8242), anti-p-p65 (Cell Signaling Technology, 3033S), anti-TFAM (Cell Signaling Technology, 8076S), and anti-β-actin (Sigma, A5316) overnight at 4°C. The binding of primary antibodies was visualized with goat anti-rabbit horseradish peroxidase (HRP)-conjugated secondary antibody (Jackson Laboratory, 115-035-033) or goat anti-mouse HRP-conjugated secondary antibody (Jackson Laboratory, 111-035-003).

### RNA-seq and real-time PCR

Total RNA was isolated by RNA-Quick Purification Kit (RN001). Then, purified total RNA was submitted to Gene Denovo Biotechnology Co. for RNA-seq.

Purified total RNA was reverse-transcribed by HiScript 1st Strand cDNA Synthesis Kit (Vazyme, R323-01). Real-time PCR was performed by SYBR premix (Roche) with the procedure: 95°C for 5 min followed by 40 3-step cycles of 95°C for 30 sec, 60°C for 15 sec, and 72°C for 20 sec. The primers used for real-time PCR were designed as follows: *Cav1* forward: 5′-AGCAAAAGTTGTAGCGCCAG-3′, reverse: 5′-GACCACGTCGTCGTTGAGAT-3′; *Ap2b1* forward: 5′-CTGGTCCAACAGGTCTTGAGCT-3′, reverse: 5′-CCACTTCTTTGGCTGTCACAGG-3′; *Rela* forward: 5′-AGGCTTCTGGGCCTTATGTG-3′, reverse: 5′-TGCTTCTCTCGCCAGGAATAC-3′; *Tnfa* forward: 5′-AAGCCTGTAGCCCACGTCGTA-3′, reverse: 5′-GGCACCACTAGTTGGTTGTCTTTG-3′; *Il1b* forward: 5′-TGCCACCTTTTGACAGTGATG-3′, reverse: 5′-TGATGTGCTGCTGCGAGATT-3′; *Nlrp3* forward: 5′-TCACAACTCGCCCAAGGAGGAA-3′, reverse: 5′-AAGAGACCACGGCAGAAGCTAG-3′; *Tfam* forward: 5′-GAGGCAAAGGATGATTCGGCTC-3′, reverse: 5′-CGAATCCTATCATCTTTAGCAAGC-3′; *Actb* forward: 5′-CATCCGTAAAGACCTCTATGCCAAC-3′, reverse: 5′-ATGGAGCCACCGATCCACA-3′. Relative gene expression was calculated using ΔΔCt by normalizing to the reference gene.

### Quantification of mtDNA copy number

MtDNA copy number in microglia were determined by mtDNA/nuclear DNA ratio according to the previous method (Quiros et al., 2017). Briefly, total DNA was isolated using the TIANcombi DNA Lyse&Det PCR Kit (TIANGEN, KG201101X). Real-time PCR was performed by SYBR premix (Roche) with the procedure: 95°C for 5 min followed by 30 3-step cycles of 95°C for 30 sec, 60°C for 15 sec, and 72°C for 20 sec. The primers used for real-time PCR were designed as follows: *ND1* forward: 5′-CTAGCAGAAACAAACCGGGC-3′, reverse: 5′-CCGGCTGCGTATTCTACGTT-3′; *16S rRNA* forward: 5′-CCGCAAGGGAAAGATGAAAGAC-3′, reverse: 5′-TCGTTTGGTTTCGGGGTTTC-3′; *HK2* forward: 5′-GCCAGCCTCTCCTGATTTTAGTGT-3′, reverse: 5′-GGGAACACAAAAGACCTCTTCTGG-3′. mtDNA copy number was calculated using the following formula: ΔCt = Ct (*ND1* or *16S rRNA*)—Ct (*HK2*). The ΔΔCt was calculated by using the mean ΔCt value of the normal microglia.

### Immunofluorescence and immunocytochemistry

Tissue sections (40 μm) and cultured cells were fixed with 4% paraformaldehyde and then permeabilized with 0.3% Triton X-100. After being blocked by 10% donkey serum, the samples were probed with anti-Iba1 (Abcam, ab5076), anti-AQP4, anti-Laminin (Abcam, ab11575), anti-NRF1, anti-NLRP3, or anti-ASC antibodies. The binding of primary antibodies was visualized with Alexa Fluor 555-conjugated donkey anti-rabbit IgG (Thermo, A31572), Alexa Fluor 488-conjugated donkey anti-mouse IgG (Thermo, A21202), or Alexa Fluor 488-conjugated donkey anti-goat IgG (Abcam, ab150133). Then, the samples were counterstained with DAPI (Thermo). Leica THUNDER Imagers or a Leica SP8 confocal microscope were used to capture the images.

### ChIP assay

ChIP experiments were performed using the SimpleChIP® Enzymatic Chromatin IP Kit (Magnetic Beads) (Cell Signaling Technology, #9003), as described previously (Wang et al., 2021). Microglia were fixed in culture medium containing 1% formaldehyde to cross-link proteins and DNA, and then were lysed and digested with micrococcal nuclease to 200–500 bp. The mixture was then immunoprecipitated with an anti-NRF1 antibody or IgG to enrich the bound DNA. The purified DNA was amplified by real-time PCR with the procedure: 95°C for 5 min followed by 40 3-step cycles of 95°C for 30 sec, 60°C for 15 sec, and 72°C for 20 sec. The primers used for real-time PCR were designed as follows: *Tfam (–668/–570)* forward: 5′-TGTACAACGTGCGCTAGGAT-3′, reverse: 5′-CCAAGATTTTCGGCTCTGCC-3′; *Rela (–879/–376)* forward: 5′-AGTGGGAGGGGCGTAACTAT-3′, reverse: 5′-ACAGGCCTTAGGGTAGAGGG-3′; *Cav1 (–1101/–993)* forward: 5′-AGCCCTGGGATTCCTCTTCA-3′, reverse: 5′-GCAAACACTCCCAATGCACA-3′; *Ap2b1 (–1426/–1282)* forward: 5′-AATACCACGCATGGGGCTCT-3′, reverse: 5′-CAATCATCCGGTCTACCCTAGC-3′; *Ap2b1 (–33/136)* forward: 5′-CAAGAAACGTGTCGCTCGAA-3′, reverse: 5′-GGGTACGGTTTCGGTCACAA-3′. Relative enrichments of target genes were calculated using ΔCt by normalizing to the IgG group.

### Dual-luciferase reporter assay

Fragments of mouse *Cav1 (–1101/–993), Ap2b1 (–1426/–1282)*, or *Ap2b1 (–33/136)* were cloned into the *Kpn*I and *Hind*III restriction sites of the pGL3-Enhancer vector. All constructs were verified by sequencing. The day before transfection, HEK293T cells were cultured on a 24-well plate in antibiotic-free culture medium. Lipofectamine 2000 reagent was used to co-transfect cells with 250 ng reporter plasmids, 7 pmol NRF1 siRNA, and 10 ng Renilla reporter plasmid (pRL-TK, Promega) as an internal control. Luciferase activities were measured by the Dual-Luciferase Reporter Assay System (Promega). Firefly luminescence signal was normalized by Renilla luminescence signal.

### Statistical analysis

GraphPad Prism v8 (GraphPad) was used to analyze the data, which included Student's *t*-test, one-way analysis of variance (ANOVA), and two-way ANOVA followed by Tukey's multiple comparisons. All the data were presented as mean ± SEM for *in vivo* experiments or mean ± SD for *in vitro* experiments. The level of significance was determined as follows: *P < 0.05, **P < 0.01, and ***P < 0.001. n.s. indicates no significance.

## Supplementary Material

mjac036_Supplemental_Files
