# Supplementary material for: NRF1-mediated microglial activation triggers high-altitude cerebral edema
Source: J Mol Cell Biol. 2022 Jun 15;14(5):mjac036. doi: 10.1093/jmcb/mjac036 (PMC9486928; doi:10.1093/jmcb/mjac036)
Supplement: mjac036_Supplemental_Files [file mjac036_supplemental_files.zip › JMCB-2021-0296.R1_Supplementary material.pdf]

Supplementary material

NRF1-mediated microglial activation triggers high-altitude cerebral edema

Xueting Wang[1,2,*], Guijuan Chen[1,2], Baolan Wan[1,2], Zhangji Dong[2,3], Yan Xue[1,2], Qianqian Luo[1,2], Dan Wang[1,2], Yapeng Lu[1,2], and Li Zhu[1,2,*]

[1] Institute of Special Environmental Medicine, Nantong University, Nantong 226019, China

[2] Co-Innovation Center of Neuroregeneration, Jiangsu Key Laboratory of Neuroregeneration, Nantong University, Nantong 226019, China

[3] Key Laboratory of Neuroregeneration of Jiangsu and Ministry of Education, Nantong University, Nantong 226019, China

* Correspondence to: Xueting Wang, E-mail: wangxueting@ntu.edu.cn; Li Zhu, Tel: +8613962988532, Fax: +86513855003376, E-mail: zhulizhou@ntu.edu.cn

Supplementary Figures

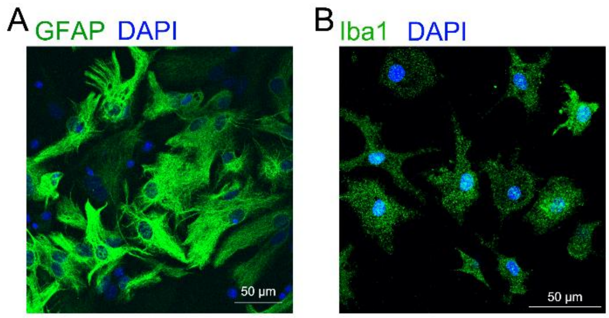

Supplementary Figure S1 Identifies of primary astrocytes and microglia. Isolated astrocytes and microglia were stained with an anti-GFAP (**A**) and anti-Iba1 antibody (**B**), respectively. Cells were counterstained with DAPI.

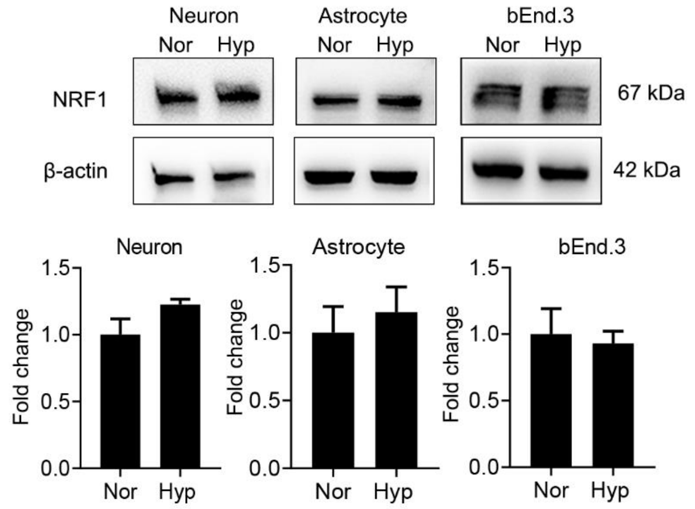

Supplementary Figure S2 NRF1 expression in neuron, astrocyte, and bEnd.3 after hypoxia treatments. Primary neuron, primary astrocyte, and bEnd.3 cells were lysed for Western blot. Protein levels of NRF1 were quantified by Image J software (Student's *t*-tests).

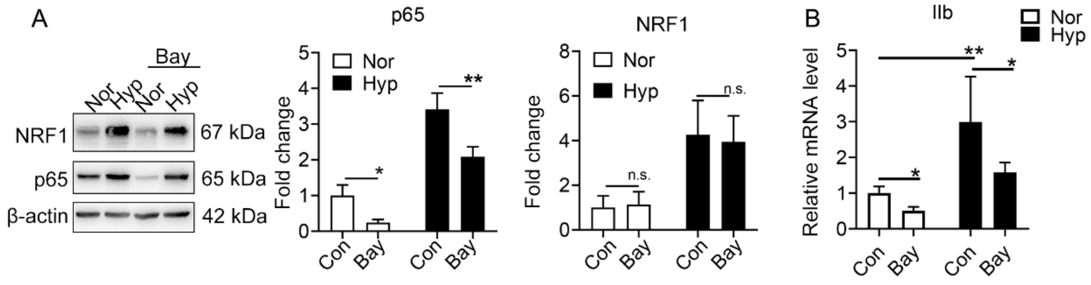

Supplementary Figure S3 NRF1 was not affected by Bay 11-7082 under both normoxia and hypoxia. Primary microglia were pretreated with Bay 11-7082 for 24 h followed by hypoxia treatment for 24 h. (**A**) Cells were lysed for NRF1 and p65 measurement by Western blot. (**B**) mRNA level of *Il1b* was quantified by real-time PCR (**P* < 0.05 and ***P* < 0.01 by two-way ANOVA).

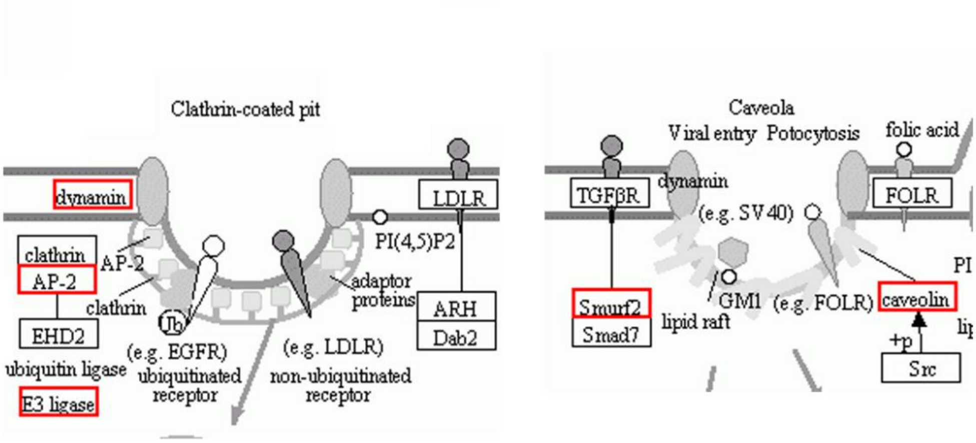

Supplementary Figure S4 Potential target genes of NRF1 in endocytosis. Genes with red border indicated the potential target genes of NRF1 by CHIP-seq.
